# Counteract Anhedonia! Introducing an Online-Training to Enhance Reward Experiencing – A Pilot Study

**DOI:** 10.32872/cpe.13751

**Published:** 2024-06-28

**Authors:** Cara Limpächer, Tordis Kindt, Jürgen Hoyer

**Affiliations:** 1 Behavioral Psychotherapy, Faculty of Psychology, Technische Universität Dresden, Dresden, Germany; 2 University Clinic and Outpatient Clinic for Psychiatry, Psychotherapy and Psychosomatics, University Hospital Halle (Saale), Halle, Germany; Philipps-University of Marburg, Marburg, Germany

**Keywords:** depression, reward experience, behavioral activation, savoring, gratitude writing

## Abstract

**Background:**

Anhedonia is a risk factor for a severe course of depression but is often not adequately addressed in psychotherapy. This study presents the Training to Enhance Reward Experience (T-REx), a novel self-help approach that uses savoring and mental imagery to target impairments in reward experience associated with anhedonia. We aimed to examine feasibility and acceptability of T-REx and exploratively investigated its effects on anhedonia and other clinical variables.

**Method:**

In an online, randomized controlled trial, 79 subjects participated for five days in T-REx or the active control condition Gratitude Writing (GW). We assessed changes in anhedonia, depression, and active behavior at inclusion, after the waiting period, post-intervention and at follow-up. The intervention effects were examined for the full sample and an anhedonic sub-sample.

**Results:**

T-REx and GW were equally feasible and clearly accepted by the sample. Both interventions significantly reduced depressive symptoms and increased behavioral activation. Although there was no significant main effect of the interventions, between-group differences were observed for depressive symptoms and active behavior at post-intervention and follow-up, favoring T-REx. Further, within-group changes for T-REx were larger than for GW. The observed effects had a greater magnitude in the anhedonic sub-sample, suggesting that individuals with more pronounced anhedonic symptoms derived greater benefit from the interventions.

**Discussion:**

This first study of T-REx provides promising results that should prompt further investigations of T-REx in clinical samples. The results suggest that T-REx has a positive effect on depression symptoms and active behavior. Further, its potential as a valuable adjunct to behavioral activation interventions is discussed.

## Highlights

- We theoretically deduced, developed, and examined a novel treatment option for anhedonia based on savoring and mental imagery.
- The Training to Enhance Reward Experience (T-REx) reduces depression and increases active behavior.
- T-REx proves to be a promising extension of behavioral activation or other CBT treatments.

Depression is one of the most frequent mental disorders worldwide and among the three leading causes of non-fatal health loss and years lived with disability (GBD 2017 Disease and Injury Incidence and Prevalence Collaborators, 2018). Psychotherapeutic approaches including cognitive-behavioral therapy are generally successful and recommended in treatment guidelines (e.g., National Institute for Health and Care Excellence [NICE], 2022). However, patients often experience relapses (36 – 54%, Steinert et al., 2014) and residual symptoms like sleep problems, fatigue, and loss of interest endure even after other symptoms of depression have already subsided (Nierenberg, 2015). Problems of reward experience and loss of interest are frequent. Of those affected by major depressive disorder, up to 75% report to suffer from anhedonia, i.e., inability to experience pleasure or enjoyment from activities that would normally be pleasurable (Franken et al., 2007). Further, 37% endure severe, chronic anhedonic symptoms, which coincide with higher severity of depression (Pelizza & Ferrari, 2009). Higher anhedonia predicts a poor longitudinal course of depression (Kessler et al., 2017). It is postulated to be a predictor of suicidal ideation (Ducasse et al., 2018) and the presence of anhedonia might ease the progression from ideation to action (Auerbach et al., 2022).

Most conceptualizations of anhedonia converge upon three main subcomponents: (1) Anticipatory pleasure represents the motivation to expend effort for rewards and includes implicit and explicit wanting processes, (2) consummatory pleasure refers to the responsivity towards rewards, (3) reward learning is defined as probabilistic and reinforcement learning of stimulus-reward contingencies (Craske et al., 2019). The dysfunction of any of the three components of the reward process may lead to the disruptive effects that anhedonia may have for psychotherapy (Rømer Thomsen et al., 2015; Wang et al., 2021).

In recent decades, psychotherapy research has primarily focused on developing strategies to reduce psychopathology, such as sadness and anxiety. Improvements in well-being are often viewed as by-products of symptom reduction. However, patients indicate the restoration of positive affect as their primary treatment goal (Demyttenaere et al., 2015). Despite the growing interest in Positive Psychology (Seligman et al., 2006), treatment options aiming to re-establish positive affect are scarce (Boumparis et al., 2016; Sandman & Craske, 2022). On a positive note, current treatment programs can likely be improved by incorporating techniques that focus on positive affect (Dunn et al., 2020). In view of the reduced responsiveness of reward processing in anhedonic patients, recent approaches suggest improving activation and mood by targeting the reward system more specifically (Forbes, 2020; Nagy et al., 2020). To this end, we want to introduce the Training to Enhance Reward Experience (T-REx), a new self-help approach that focuses on restoring and actively creating positive affect.

## Training to Enhance Reward Experience

The Training to Enhance Reward Experience (T-REx) was derived from the literature on mechanisms underlying anhedonia. It takes an integrative approach and incorporates techniques such as savoring and mental imagery, that have already been shown to be effective in increasing positive affect. Savoring is a meta-cognitive process that refers to the process of “generating, intensifying, and prolonging enjoyment through one’s own volition” (Bryant, 2003, p. 176). It describes the ability to regulate positive emotions by looking forward to an upcoming positive event, savoring the moment while the positive event takes place and looking back on positive experiences (Bryant, 1989). The process of savoring intensifies and prolongs the experienced pleasure and reward. In the training, mental imagery is used to facilitate savoring of positive experiences in the past and also to vividly anticipate positive moments and emotions elicited by positive experiences in the future. The training is not a novel intervention per se but rather a new approach that is intended to be simple, effective within a short period of time, and a valuable adjunct to existing interventions, notably Behavioral Activation (BA) treatments (e.g., Hoyer & Vogel, 2018).

## Purpose of the Present Study

In a pilot randomized controlled trial we compared T-REx with Gratitude Writing (GW), an empirically supported positive psychology intervention known for enhancing positive affect and well-being (Jans-Beken et al., 2020). The study focused on assessing the effectiveness, acceptability, and feasibility of T-REx in comparison to GW as an active control condition. As our main research question, we wanted to examine the intervention's effect on symptoms of anhedonia, depression, and active behavior. While taking part in T-REx, participants train the ability to savor positive moments in the past, when they regularly and repeatedly reminisce about positive experiences. Consequently, the positive affect that has been felt is intensified and prolonged to counteract anhedonic and depressive symptoms. Therefore, we expected that participation in the T-REx group results in a significantly greater reduction of anhedonic and depressive symptoms than in the GW group, across all measurement points. Further, we expected a significantly greater increase in active behavior for T-REx compared to GW, because mental imagery of future activities has shown to increase the motivation to actually engage in those activities (Heise et al., 2022). Additionally, we expected improvements in anhedonia, depression, and active behavior for both groups from pre- to post-intervention, but not during the waiting period.

## Method

### Sample

Participants were recruited through online forums and websites with a focus on depression, psychology, or mental health, and in lectures at several universities in Germany. Inclusion criteria were an age between 18 and 65 years, being able to write using a PC, and having at least good German language skills. Exclusion criteria were obsessions or compulsions, acute suicidality, psychotic symptoms, substance abuse, currently receiving psychotherapeutic counseling, started or changed dose of antidepressant medication during the past 3 months. All inclusion and exclusion criteria were assessed using single items. Informed consent was obtained before participation, and the study was conducted in accordance with the declaration of Helsinki.

### Procedure

The web-based longitudinal study was conducted online via SoSci Survey (Leiner, 2021); all questionnaires and instructions for the interventions were delivered on this platform. Directly after study inclusion, participants completed the baseline questionnaire (t1). We randomly assigned participants to either T-REx or GW. Both groups were compared to a within patient waitlist control group. After a waiting time of one week the respective intervention started and participants completed the pre-intervention questionnaires (t2). After participating in the interventions for five days, participants filled in post-intervention questionnaires at the last intervention day (t3) and follow-up questionnaires two weeks afterwards (t4).

### Material

#### Interventions

Both interventions were designed as online self-guided approaches. For comparability, the time required for the interventions was similar, with both interventions taking approximately 15 minutes per day. Each day of the intervention, participants received an email with a link that took them directly to the intervention’s instructions, provided in both written and audio format.

T-REx consists of four parts. The initial phase on day one consists of psychoeducational information about the training rationale (*reward sensitization)*. The latter three parts of the training target components of the reward system and thus aim to enhance reward experience by building on the three time orientations of savoring. Participants are encouraged to focus their attention on experiences (activities, perceptions, etc.) that they perceive as pleasant. Therefore, their task for the next days is to collect positive moments in everyday life. In the standardized instructions, we provide two examples of methods for collecting these moments (e.g., a smartphone to take a picture of something representative of the experience), however, participants are free to choose their own method (*reward registration*). Each evening, participants are asked to recall the positive moments they collected during the day and to reminisce about them by mentally visualizing these moments (*reward reliving*). Lastly, participants are asked to think of positive experiences that could occur the next day (*reward anticipation*). Audio-instructions for positive mental time travel are used as reinforcing enjoyment experience strategies for recalling and anticipating rewards. Instructions for mental imagery were adapted from Renner et al. (2019). We opted for mental imagery as it has demonstrated superior effectiveness as a motivational amplifier in activity scheduling when compared to verbal reasoning, as evidenced by Ji et al. (2021).

The comparator intervention GW consists of two parts. Similar to T-REx, participants receive a psychoeducational introduction to the intervention on the first day. Every evening on the following four days, subjects receive an instruction to write about something they are grateful for. Within this exercise, gratitude can be directed to people as well as to experiences, situational circumstances, or other personal topics. Once participants identify something they are grateful for, they are instructed to write about it in as much detail as possible, including any feelings or thoughts that arise. The instructions for GW have been developed after reviewing the instructions of Magyar-Moe (2009) and Rupp et al. (2018).

#### Primary Outcome Measures

We used the Snaith-Hamilton-Pleasure-Scale (SHAPS; Snaith et al., 1995; German version: Franz et al., 1998) to assess anhedonic symptoms. The questionnaire consists of 14 items and subjects are instructed to imagine whether they might feel pleasure during certain experiences. Snaith et al. proposed to recode the four response categories into dichotomous categories, that is, agree and disagree (score 0 and 1). More recent papers have used a continuous scoring method to increase sensitivity to change (Franken et al., 2007), producing scores ranging from 14 (not at all anhedonic) to 56 (severely anhedonic). The present study adopts this continuous scoring approach. The internal reliability of the continuously scored SHAPS has been found to be adequate in both non-clinical (α = 0.91) and clinical (α = 0.94) samples (Franken et al., 2007). The internal reliability for the continuously scored SHAPS was also adequate in the current sample (α = 0.79).

The Beck’s Depression Inventory II (BDI-II; Beck et al., 1996; German version: Hautzinger et al., 2006) is a widely used questionnaire in both clinical and non-clinical samples, includes 21 items that can be rated on a 4-point scale (0 – 3) and assesses somatic-affective and cognitive dimensions of depression. The total score can range between 0 and 63 and indicates mild (≥ 16), moderate (20-28) or severe (≥ 29) depressive symptoms. Psychometric properties and validity are well-established (Herzberg et al., 2008; Kühner et al., 2007), the BDI-II showed high internal consistency (α = .92-.93) and high test-retest reliability (*r* = .93, Beck et al., 1996), comparable to this study (α = .95).

The 9-item short form of the Behavioral Activation for Depression Scale – short form (BADS; Manos et al., 2011; German version: Teismann et al., 2016) assesses concepts of BA (activity and avoidance) by measuring behavioral activity in the past week with statements that can be rated on a 7-point scale ranging from 0 (not at all) to 6 (completely). The summated score ranges from 0 to 54 and higher scores refer to greater activity (Kanter et al., 2007). The BADS showed good internal consistency in previous studies (α = .85, Teismann et al., 2016), and in this study (α = .80).

The primary outcome measures reported here differ from those outlined in the preregistration, where initially more outcome measures were planned. Due to space constraints, we opted to report only the most pertinent outcomes.

#### Additional Measures

To examine acceptability, we applied a feedback questionnaire with three items that were rated on a 4-point scale (adapted from Robichaud et al., 2020). Participants provided feedback on their overall satisfaction with the intervention, the quality of the study material, and whether the time effort was worth it. A fourth item asked participants if they would recommend the intervention to a friend who suffers from loss of pleasure or interest.

To address the feasibility of the interventions we examined retention and attrition rates, as measured by the percentage of dropout between baseline (t1) and follow-up (t4). We separately assessed adherence rates as measured by the relative number of subjects who completed all five days of the respective intervention. To account for potential attrition bias (Dumville et al., 2006), we included comparisons of baseline characteristics between dropouts and completers using *t*-tests for continuous variables and chi-square tests of independence for categorical variables.

### Statistical Analysis

Intent-to-treat analyses were performed using multilevel modeling (MLM), assuming data were missing at random. As one cannot prove that data is missing at random, we first examined whether participants with missing data differed from those with complete data on any demographic or pretreatment level of the study variables. A linear mixed model for each of the three outcome measures was implemented with a random intercept for subject. The models included SHAPS-score, BDI-II score, or BADS-score, respectively, as the outcome variable, the level 1 predictor time (t1, t1, t3, t4), the level 2 predictor group (T-REx vs. GW), and a cross-level interaction between time and group. We tested the interaction of time and group by comparing the full to reduced models without the respective interaction term via likelihood ratio tests (LRTs). We specified T-REx and baseline measurement (t1) as reference categories and parameters were estimated with the maximum likelihood estimation method. Differences from t1 to t2 represent waiting time, differences from t1 to t3 post-intervention differences and from t1 to t4, the follow-up-period. Estimated marginal means, planned contrasts, within and between-group effect sizes (expressed as Cohen’s *d*), and confidence intervals (CIs) were derived from the mixed-modeling analysis. In accordance with Cohen (1988), effect sizes of *d* = 0.2 were interpreted as small, of *d* = 0.5 as medium and of *d* ≥ 0.8 as large.

In all analyses α was set to .05. We used R (R Core Team, 2023) with the following packages: lme4 (Bates et al., 2015) to perform a linear mixed effects analysis, and emmeans (Lenth et al., 2022) to calculate the statistical significance of pairwise differences.

## Results

### Participant Flow and Characteristics

In total, 251 individuals attempted to participate in the study. Of these, 172 were excluded as ineligible or declined to participate. Following the screening, 79 participants (65 females, *M*_age_[*SD*] = 26.44[9.31]) in total were randomized to either T-REx (*n* = 39) or GW (*n* = 40) and provided baseline data (see Figure S1 for CONSORT flowchart in the online Supplementary Materials). Table S1 in the Supplementary Materials shows the demographic and clinical characteristics. At baseline, the groups did not significantly differ in demographic characteristics and clinical variables (all *p*s > .05, see Table S1 in the Supplementary Materials).

### Feasibility and Acceptability Analysis

The overall retention rate from baseline to follow-up was 72% (*n* = 57), thus *n* = 22 subjects dropped out of the study before completing the last assessment. Looking at T-REx and GW separately, the retention rates (including follow-up) were 82% (*n* = 32) and 63% (*n* = 25), respectively. The dropout was higher for GW than for T-REx, although this difference was not statistically significant χ^2^ = 3.76, *p* = .053. Adherence for T-REx was 90% and 75% for GW. To identify potential baseline factors that might have affected whether participants dropped out by the two weeks follow-up, and whether this varied between interventions, a series of factorial ANOVAs (group by dropout) were conducted. Participants who dropped out were on average older than completers (*M* = 31.8 vs. 24.4, *F*[1,75] = 12.88, *p* < .001, $\eta_{p}^{2}$ = < 0.01), but this did not differ by group, with a non-significant interaction (*F*[1, 75]= 0.006, *p* = .938, $\eta_{p}^{2}$ < 0.01). There were no differences on clinical outcomes or demographic variables for those who dropped out by two weeks follow-up, nor interactions with group (all *p*s > .05).

On average, participants indicated their satisfaction regarding the interventions, quality of study materials, and time effort between 3,00 – 3,29 from 4. All subjects who completed T-REx would recommend the intervention to a friend suffering from a loss of pleasure or interest. Within completers of GW 90% would recommend the intervention to a friend, although this difference was not statistically significant (χ^2^ = 3.67, *p* = .055).

### Changes in Anhedonia, Depression, Behavioral Activation

We compared the mixed effect models with and without the interaction term for all primary outcome measures. For all three outcome measures the null-hypothesis could not be rejected: The models with the interaction term did not explain significantly more variance than the reduced models (SHAPS: χ^2^[3] = 1.14, *p* = .768; BDI-II: χ^2^[3] = 2.80, *p* = .423; BADS: χ^2^[3] = 2.58, *p* = .461), hence there was no significant difference in average slope between the two groups. Since the interaction of time and group was not significant, we consider main effects of time and group in the following.

As predicted, the change in SHAPS, BDI-II and BADS-score over the one week waiting time was small and not significant (all *p*s > .05). There was no significant main effect of time or group on the SHAPS-score at any of the measurement occasions (all *p*s > .05). However, we found a significant main effect of time for BDI-II at post-training (β = -3.12, *SE* = 0.96, *p* = .001) and follow-up (β = -3.96, *SE* = 0.99, *p* < .001), meaning that compared to baseline, after the intervention and at two weeks follow-up, BDI-II in both groups was roughly three to four points lower than at baseline. We found no significant main effect of group (β = 2.65, *SE* = 2.41, *p* = .275), while data inspection revealed that participants in T-REx have on average three BDI-II points less than participants in GW. Again, we found a significant main effect of time for BADS at post-training (β = 4.05, *SE* = 1.22, *p* = .001) and follow-up (β = 5.65, *SE* = 1.26, *p* < .001), indicating that after both interventions and at two weeks follow-up, BADS scores were on average roughly four to five points higher than at baseline. Further, we found no main effect of group on BADS-scores (β = -0.66, *SE* = 2.16, *p* = .757). In addition, we rerun the analyses excluding the items of the BDI-II that Cogan et al. (2024) recently identified as assessing anhedonia (items 4, 12, 15, 21). However, results for the models with and without the interaction term and for within and between group changes were only marginally different, when this reduced version of the BDI was used.

Observed and estimated marginals means based on the multilevel models, as well as contrasts and between-group effect sizes are presented in Table 1. Note that the confidence interval of Cohen's *d* includes zero for all between-group effect sizes.

**Table 1:** Means and Standard Deviations/Standard Errors for Observed and Estimated Data, Contrasts and Cohen’s d for the Full Sample

Outcome	Observed		Estimated		Contrast [95% CI]	*d*
	*M* (*SD*)		*M* (*SE*)			
	T-REx	GW	T-REx	GW		
SHAPS						
t1	24.4 (5.97)	24.7 (8.46)	24.4 (1.07)	24.7 (1.05)	-0.32 [-3.28, 2.65]	-0.07
t2	24.9 (6.39)	23.9 (6.58)	24.9 (1.07)	23.9 (1.05)	0.93 [-1.99, 3.94]	0.23
t3	24.1 (5.28)	23.1 (7.00)	23.8 (1.10)	22.9 (1.15)	0.87 [-2.28, 4.02]	0.20
t4	23.5 (5.06)	22.8 (7.65)	23.2 (1.13)	23.0 (1.22)	0.14 [-3.14, 3.43]	0.03
BDI-II						
t1	11.2 (8.06)	13.8 (13.7)	11.18 (1.74)	13.82 (1.72)	-2.65 [-7.50, 2.21]	-0.65
t2	11.0 (7.89)	14.0 (13.0)	11.03 (1.74)	14.03 (1.72)	-3.00 [-7.85, 1.85]	-0.74
t3	8.57 (6.77)	10.7 (11.7)	8.06 (1.76)	10.98 (1.78)	-2.92 [-7.88, 2.04]	-0.72
t4	7.19 (5.98)	11.5 (14.0)	7.22 (1.78)	12.15 (1.82)	-4.93 [-9.97, 0.12]	-1.21
BADS						
t1	30.8 (8.71)	30.1 (11.0)	30.8 (1.56)	30.1 (1.54)	0.67 [-3.66, 5.00]	0.13
t2	31.6 (8.72)	30.0 (10.6)	31.6 (1.56)	30.0 (1.54)	1.59 [-2.74, 5.92]	0.31
t3	34.1 (8.24)	33.2 (10.6)	34.8 (1.59)	32.9 (1.64)	1.91 [-2.61, 6.42]	0.37
t4	35.7 (6.86)	33.4 (11.9)	36.4 (1.62)	32.8 (1.71)	3.65 [-1.00, 8.31]	0.71

*Note.* T-REx = Training to Enhance Reward Experience; GW = Gratitude Writing; SHAPS = Snaith-Hamilton-Pleasure-Scale; BDI-II = Beck’s Depression Inventory II; BADS = Behavioral Activation for Depression Scale; t1 = baseline (*n* = 79); t2 = pre-intervention (*n* = 79); t3 = post-intervention (*n* = 65); t4 = two-week follow-up (*n* = 57).

### Exploratory Analysis in Anhedonic Sub-Sample

The participants from this study were recruited from the general population. However, since we focused on including patients with self-reported depressive symptoms, some individuals in the sample exhibited stronger anhedonic symptoms. To see whether the interventions would be effective if anhedonia is more severe, we carried out the same analyses for an anhedonic sub-sample. Subjects with a cut-off score ≥ 2 in the SHAPS original coding at baseline, were classified as anhedonic. We decided to use a slightly more liberal cut-off score than recommended by Snaith et al. (1995) to ensure an adequate sample size for analysis. Given the complexity and multifaceted nature of anhedonia, a stringent cut-off could have led to the exclusion of individuals who still exhibit clinically relevant symptoms, albeit to a lesser extent. This criterion applied to *n* = 17 (44%) subjects in the T-REx group and *n* = 18 (45%) in the GW group. The following analyses were based on the anhedonic sub-sample (*n* = 35, 28 females, *M*_age_[*SD*] = 26.09[8.25]).

**Figure 1:**
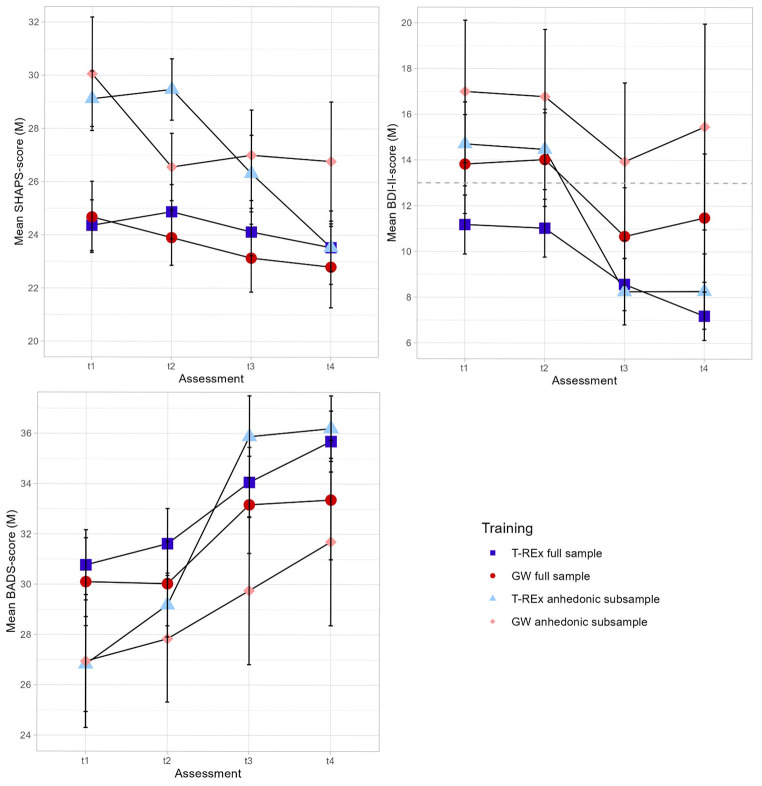
Mean Values of BDI-II, SHAPS and BADS for Each Assessment in Both Groups (T-REx, GW) for the Total Sample (*N* = 79) and Anhedonic Sub-Sample (*n* = 36) *Note.* T-REx = Training to Enhance Reward Experience; GW = Gratitude Writing; SHAPS = Snaith-Hamilton-Pleasure-Scale; BDI-II = Beck’s Depression Inventory II; BADS = Behavioral Activation for Depression Scale; cut-off scores for BDI-II (total score of > 13 = mild depression, Beck et al., 1996) included as dotted line.

Neither for SHAPS, BDI-II, nor BADS the models with the interaction term (time x group) explained significantly more variance than the reduced models (all *p*s > .05). Therefore, there was no significant difference in the average slope between the two groups. Since the interaction of time and group did not yield significance, we focus on the main effects of time and group in subsequent analyses. Regarding SHAPS, in the anhedonic sub-sample, the main effect of time was not significant at post-training (β = -2.69, *SE* = 1.73, *p* = .124) but significant at follow-up (β = -5.51, *SE* = 1.77, *p* = .002). We found significant effects of time on BDI-II at post-training (β = -6.41, *SE* = 1.67, *p* < .001) and follow-up (β = -5.99, *SE* = 1.71, *p* < .001). Likewise, we found a main effect of time on BADS at post-intervention (β = 8.75, *SE* = 1.78, *p* < .001) and follow-up (β = 9.08, *SE* = 1.82, *p* < .001). Figure 1 shows the mean values of all measures for baseline, pre-intervention, post-intervention, and follow-up for the full sample and the anhedonic sub-sample respectively. Observed and estimated marginals means based on the multilevel models, as well as contrasts and between-group effect sizes for the anhedonic sub-sample are presented in Table 2. We found large between-group effect sizes for BDI-II and BADS scores at post-intervention and at follow-up (all *d*s > .80). Note that the CI includes zero for all contrasts and *d*s presented in Table 2. Within-group effect sizes for the full sample and the anhedonic sub-sample are depicted in Table 3.

**Table 2:** Means and Standard Deviations/Standard Errors for Observed and Estimated Data, Contrasts and Cohen’s d for the Anhedonic Sub-Sample

Outcome	Observed		Estimated		Contrast [95% CI]	*d*
	*M* (*SD*)		*M* (*SE*)			
	T-REx	GW	T-REx	GW		
SHAPS						
t1	29.1 (4.30)	30.1 (9.05)	29.1 (1.54)	30.1 (1.49)	-0.94 [-5.19, 3.31]	-0.19
t2	29.5 (4.76)	26.6 (5.37)	29.5 (1.54)	26.6 (1.49)	2.92 [-1.33, 7.16]	0.59
t3	26.3 (5.76)	27 (6.80)	26.4 (1.58)	27.0 (1.57)	-0.56 [-4.97, 3.85]	-0.11
t4	23.5 (5.34)	26.8 (8.07)	23.6 (1.62)	26.8 (1.71)	-3.21 [-7.87, 1.45]	-0.65
BDI-II						
t1	14.7 (7.58)	17 (13.2)	14.71 (2.60)	17.00 (2.53)	-2.29 [-9.57, 4.98]	-0.48
t2	14.5 (7.26)	16.8 (12.5)	14.47 (2.60)	16.78 (2.53)	-2.31 [-9.58, 4.97]	-0.49
t3	8.25 (5.79)	13.9 (13.8)	8.30 (2.62)	13.34 (2.57)	-5.04 [-12.41, 2.33]	-1.06
t4	8.27 (6.37)	15.5 (16.2)	8.72 (2.65)	15.22 (2.66)	-6.50 [-14.01, 1.02]	-1.37
BADS						
t1	26.8 (7.78)	26.9 (11.2)	26.8 (2.20)	26.9 (2.14)	-0.12 [-6.24, 6.00]	-0.02
t2	29.2 (5.23)	27.8 (10.7)	29.2 (2.20)	27.8 (2.14)	1.34 [-4.78, 7.47]	0.26
t3	35.9 (6.5)	29.8 (11.8)	35.6 (2.23)	30.2 (2.20)	5.34 [-0.91, 11.59]	1.05
t4	36.2 (5.05)	31.7 (12.0)	35.9 (2.26)	31.4 (2.31)	4.49 [-1.95, 10.94]	0.89

*Note.* T-REx = Training to Enhance Reward Experience; GW = Gratitude Writing; SHAPS = Snaith-Hamilton-Pleasure-Scale; BDI-II = Beck’s Depression Inventory II; BADS = Behavioral Activation for Depression Scale; t1 = baseline (*n* = 35); t2 = pre-intervention (*n* = 35); t3 = post-intervention (*n* = 32); t4 = 2-weeks follow-up (*n* = 28).

**Table 3:** Within-Effect Sizes (Cohen’s d) and 95% CIs for the Full Sample and the Anhedonic Sub-Sample

Outcome	Condition	Within-group *d*s [95% CI] Full Sample		Within-group *d*s [95% CI] Anhedonic sub-sample	
		Baseline to post-intervention	Baseline to follow-up	Baseline to post-intervention	Baseline to follow-up
SHAPS	T-REx	0.13 [-0.34, 0.59]	0.28 [-0.21, 0.76]	0.54 [-0.17, 1.26]	**1.11** [0.38, 1.84]
	GW	0.40 [-0.09, 0.90]	0.38 [-0.15, 0.92]	0.62 [-0.09, 1.33]	0.65 [-0.11, 1.41]
BDI	T-REx	**0.77** [0.29, 1.24]	**0.97** [0.48, 1.46]	**1.35** [0.62, 2.07]	**1.26** [0.51, 2.00]
	GW	**0.70** [0.19, 1.20]	0.41 [-0.12, 0.95]	**0.77** [0.05, 1.49]	0.37 [-0.40, 1.15]
BADS	T-REx	**-0.78** [-1.26, -0.31]	**-1.09** [-1.58, -0.61]	**-1.72** [-2.45, -1.00]	**-1.79** [-2.53, -1.05]
	GW	**-0.54** [-1.04, -0.04]	-0.52 [-1.05, 0.02]	-0.65 [-1.37, 0.07]	**-0.88** [-1.65, -0.11]

*Note.* T-REx = Training to Enhance Reward Experience; GW = Gratitude Writing; SHAPS = Snaith-Hamilton-Pleasure-Scale; BDI-II = Beck’s Depression Inventory II; BADS = Behavioral Activation for Depression Scale; *d* is printed in bold if the CI does not contain 0.

## Discussion

This is the first study investigating the effects of T-REx, a new self-help intervention targeting anhedonia. In an online randomized controlled trial, we used GW, hence an empirically tested intervention, as comparison. We examined the interventions’ feasibility, acceptability, and treatment effects on anhedonia, depression, and behavioral activation – both in the full sample as well as in an anhedonic sub-sample.

Both interventions significantly reduced depressive symptoms and increased behavioral activation from baseline measurement to post intervention. The observed favorable effect persisted until the follow-up measurement and appeared to become subsequently amplified. We found medium to high between-group effect sizes in favor of T-REx for depressive symptoms and active behavior at post-intervention and at follow-up, but the main effect of intervention was not statistically significant. In the full, as well as in the anhedonic subsample, within-arm changes in the T-REx group from baseline to post-intervention and to follow-up were consistently larger than in the GW group (especially for BDI-II and BADS). The observed effects had a greater magnitude within the anhedonic sub-sample, suggesting that individuals with more pronounced anhedonic symptoms derived greater benefit from the interventions. Relatively higher retention and thus lower attrition rates in T-REx as well as a favorable adherence rate, suggests a greater preference for T-REx. Participants’ feedback on the interventions was predominantly good to excellent. Further, the dropout rates can also be interpreted in terms of acceptability (Feeley et al., 2009), thereby supporting the acceptability of both interventions.

Subsequently, a more detailed examination is conducted to explore the effects of the interventions on depression symptoms, behavioral activation, and anhedonia, respectively. After participating in the interventions, the estimated means for both groups were below the cut-off score for mild depression (BDI-II < 13, Beck et al., 1996). We found medium to large effect sizes in diminishing depressive symptoms from baseline to post-intervention and sustained through follow-up for T-REx, while only small to medium effect sizes were noted for GW. Our findings indicate that a brief 5-day intervention combining reward sensitivity training, savoring exercises, and mental imagery effectively mitigates depressive symptoms. These data corroborate findings of previous studies that showed that savoring is a protective factor for depression as higher savoring was associated with lower depressive symptoms (Chiu et al., 2020; Ford et al., 2017). Future studies are needed to observe which temporal savoring domain is likely to reduce depression symptoms most. Research so far indicates that momentary savoring has stronger negative association with depressive symptoms than do reminiscing and anticipating (Bryant, 2003; Kahrilas et al., 2020).

Behavioral activation was effectively increased even though T-REx did not include activity planning, a core element of BA (Kanter et al., 2009). This is consistent with other studies suggesting that mental imagery of activities serves as a “motivational amplifier” for engaging in activities (Ji et al., 2021; Renner et al., 2019). Hence, it is likely that T-REx, especially reward anticipation, may prompt a more active behavior, i.e., increases the motivation to engage in pleasurable activities, and that T-REx therefore has the potential to be optimally combined with BA interventions. Our results are in line with the pattern of co-occurrence of increased behavioral activation and decreased depressive symptoms previously found in response to behavioral activation interventions (Hoyer & Vogel, 2018; Limpächer et al., 2023; Melicherova et al., 2024). Albeit we did not gather data regarding the implementation of imagined activities for the subsequent day; this aspect could be explored in a future study. Such an investigation would yield an objective measure of behavioral activation, surpassing mere reliance on self-reported data.

Surprisingly, despite T-REx being specifically designed to alleviate anhedonia, the SHAPS was the sole outcome measure where no significant effect of time was observed. One potential explanation for the missing effect in the full sample, could be attributed to the low baseline scores, suggesting little room for improvement. Moreover, it is conceivable that anhedonia may require a longer time to repair, gradually resolving as depressed mood recovers and individuals consistently engage in potentially rewarding activities. The validity of this assumption is supported by the results of the anhedonic sub-sample, where a significant improvement in anhedonic symptoms was observed in both groups at follow-up. These findings align with the results reported by Alsayednasser et al. (2022), who conducted a comparison of cognitive-behavioral-therapy and BA treatments for individuals with depression: across all measurement points and for both conditions anhedonia was repaired to a lesser extent than depression.

### Limitations and Future Research

A number of methodological limitations need to be considered. First, due to the pilot nature of this study, our sample size was rather small. Thus, the design was likely underpowered, especially for the confirmation of interactions between group and time, leading to a decreased chance of detecting treatment differences. Moreover, generalizability of our findings is limited given the sociodemographic profile of our sample that is of young age and mostly female. We note that the sample was a healthy or rather subclinical sample, as, for example, no cut-off regarding anhedonia or depression was set for participation, and, because the study took place online, a detailed clinical assessment was not possible. Hence, our results provide a proof of concept and call for further replications with larger (clinical) samples.

Second, we acknowledge that the positive changes observed may be attributed to other factors than the online interventions, which include the attention of the study team, the neutral course of symptoms related to depression, or other variables that may impact symptom burden but are unrelated to T-REx or GW (e.g., stress associated with school/work, additional coping attempts).

Third, although we countered systematic bias by randomly assigning interventions, our research on primary and secondary outcomes relies on self-report measures, which are known to be prone to several types of bias, including confirmation bias, retrospective recall bias, and social desirability bias.

Naturally, new research questions arise from these limitations. Given the encouraging results, the next step should be to proceed from this pilot study to a large-scale trial. Future studies are essential to examine how well the observed effects translate to, or even increase in clinical samples and other settings (i.e., offline). Furthermore, it would be valuable to conduct a more in-depth analysis of the dose-response relationship of savored moments. This analysis would explore whether the effects of T-Rex intensify as more positive events are collected, reminisced upon, and imagined for the next day. Moreover, given that sharing positive experiences with others is considered a savoring strategy associated with greater well-being (Gable et al., 2004; Lambert et al., 2013), it seems plausible to assume that group therapies could be a particularly potent setting for implementing T-REx.

In conclusion, this study offers encouraging evidence supporting the feasibility and acceptance of T-REx as an intervention to alleviate depression symptoms and enhance behavioral activation over a brief intervention period. Nevertheless, the findings once again emphasize the challenging nature of treating anhedonia through psychotherapeutic interventions.

## Supplementary Materials

The Supplementary Materials include the following items:

- The preregistration for this study (see Limpächer et al., 2021S)
- Additional information (see Limpächer et al., 2024S):
  - A CONSORT flow chart of participants
  - A table with pretreatment and demographic characteristics of the intent-to-treat sample

LimpächerC.
KindtT.
HoyerJ.
 (2021S). Supplementary materials to "Counteract anhedonia! Introducing an online-training to enhance reward experiencing – A pilot study"
[Preregistration]. PsychOpen. https://drks.de/search/en/trial/DRKS00025758
10.32872/cpe.13751PMC1130391439119054

LimpächerC.
KindtT.
HoyerJ.
 (2024S). Supplementary materials to "Counteract anhedonia! Introducing an online-training to enhance reward experiencing – A pilot study"
[Additional information]. PsychOpen. 10.23668/psycharchives.14656
PMC1130391439119054

## Data Availability

The data that support the findings of this study are available from the corresponding author, CL, upon reasonable request.

## References

[sp1_r1] Limpächer C. Kindt T. Hoyer J. (2021 S). Supplementary materials to "Counteract anhedonia! Introducing an online-training to enhance reward experiencing – A pilot study" [Preregistration]. PsychOpen. https://drks.de/search/en/trial/DRKS00025758 10.32872/cpe.13751 PMC11303914 39119054

[sp1_r2] Limpächer C. Kindt T. Hoyer J. (2024 S). Supplementary materials to "Counteract anhedonia! Introducing an online-training to enhance reward experiencing – A pilot study" [Additional information]. PsychOpen. 10.23668/psycharchives.14656 PMC11303914 39119054

